# Molecular Dynamic Studies of the Complex Polyethylenimine and Glucose Oxidase

**DOI:** 10.3390/ijms17111796

**Published:** 2016-10-27

**Authors:** Beata Szefler, Mircea V. Diudea, Mihai V. Putz, Ireneusz P. Grudzinski

**Affiliations:** 1 Department of Physical Chemistry, Faculty of Pharmacy, Collegium Medicum, Nicolaus Copernicus University, Kurpinskiego 5, 85-096 Bydgoszcz, Poland; 2 Department of Chemistry, Faculty of Chemistry and Chemical Engineering, Babes-Bolyai University, 400028 Cluj-Napoca, Romania; diudea@gmail.com; 3 Laboratory of Structural and Computational Physical-Chemistry for Nanosciences and QSAR, Biology-Chemistry Department, West University of Timisoara, Str. Pestalozzi No. 16, 300115 Timisoara, Romania; 4 Laboratory of Renewable Energies-Photovoltaics, R&D National Institute for Electrochemistry and Condensed Matter (INCEMC), Dr. A. Paunescu Podeanu Str. No. 144, 300569 Timisoara, Romania; 5 Department of Pharmaceutical Nanotechnology and Nanotoxicology, Faculty of Pharmacy, Medical University of Warsaw, 02-097 Warsaw, Poland; ireneusz.grudzinski@wum.edu.pl

**Keywords:** polyethylenimine (PEI), 3QVR, glucose oxidase (GOx), docking, molecular dynamics

## Abstract

Glucose oxidase (GOx) is an enzyme produced by Aspergillus, Penicillium and other fungi species. It catalyzes the oxidation of β-d-glucose (by the molecular oxygen or other molecules, like quinones, in a higher oxidation state) to form d-glucono-1,5-lactone, which hydrolyses spontaneously to produce gluconic acid. A coproduct of this enzymatic reaction is hydrogen peroxide (H_2_O_2_). GOx has found several commercial applications in chemical and pharmaceutical industries including novel biosensors that use the immobilized enzyme on different nanomaterials and/or polymers such as polyethylenimine (PEI). The problem of GOx immobilization on PEI is retaining the enzyme native activity despite its immobilization onto the polymer surface. Therefore, the molecular dynamic (MD) study of the PEI ligand (C14N8_07_B22) and the GOx enzyme (3QVR) was performed to examine the final complex PEI-GOx stabilization and the affinity of the PEI ligand to the docking sites of the GOx enzyme. The docking procedure showed two places/regions of major interaction of the protein with the polymer PEI: (LIG1) of −5.8 kcal/mol and (LIG2) of −4.5 kcal/mol located inside the enzyme and on its surface, respectively. The values of enthalpy for the PEI-enzyme complex, located inside of the protein (LIG1) and on its surface (LIG2) were computed. Docking also discovered domains of the GOx protein that exhibit no interactions with the ligand or have even repulsive characteristics. The structural data clearly indicate some differences in the ligand PEI behavior bound at the two places/regions of glucose oxidase.

## 1. Introduction

Glucose oxidase (β-d-glucose: oxygen 1-oxidoreductase, GOx) is an enzyme produced by *Aspergillus*, *Penicillium* and other fungi species. It is a dimeric protein composed of two identical subunits (i.e., monomers), each showing two domains: one domain binds to the substrate (β-d-glucose) while the other domain binds non-covalently to a cofactor, namely the flavin adenine dinucleotide (FAD), a molecule acting in biological redox reactions. In the GOx enzyme, FAD works as an electron acceptor, being reduced to FADH_2_, which is finally oxidized by an electron acceptor, e.g., O_2_, which is reduced to hydrogen peroxide (H_2_O_2_). The active site of GOx contains three important amino acids involved in this catalytic reaction: His516, and Glu412, which is hydrogen-bonded to His559. Glucose oxidase is a glycoside of mannose carbohydrate (around 16%) or can be freely isolated, as a non-glycosylated enzyme, from the fungus *Phanerochaete chrysosporium*. The synthesis of GOx in fungi is promoted by O_2_, which induces the transcription of the enzyme [1,2,3]. GOx has become commercially important in the last few years, gaining important use in chemical, pharmaceutical, food, beverage, and other industries. In addition, the gluconic acid produced in the hydrolysis of d-glucono-1,5-lactone has its own important industrial uses [4]. GOx is used as a biosensor (e.g., to measure the concentration of glucose in blood, given the dramatic increase of the number of diabetics in the world). In this respect, the enzyme is immobilized (by a non-covalent bonding) on various nanomaterials and/or chemicals, e.g., carbon nanotubes (CNTs), chitosans, poly(*N*-vinyl imidazole) hydrogels, etc. [5,6,7]. Recently, a Polish group reported the immobilization of gamma-globulins from human and bovine blood and human polyclonal antibody (IgG) on carbon-encapsulated iron nanoparticles, with magnetic properties [8]. Other authors demonstrated that the activity of two enzymes (r-chymotrypsin and soybean peroxidase) dramatically decreases after their adsorption onto the surface of single-walled carbon nanotubes [9]. The aim of these works is to retain the enzyme native activity despite its immobilization on a rigid surface, e.g., of carbon nanoparticles. A recent molecular dynamics study [10] reported the interaction energy of the SWNT:PSE:GOx complex in the water environment; in parallel, the molecular dynamics performed on the above complex demonstrated the absence of strong interactions between GOx and the nanotube surface. This fact allowed supposing that GOx activity is not changed under immobilization as the SWNT:PSE:GOx complex.

**Figure 1:**
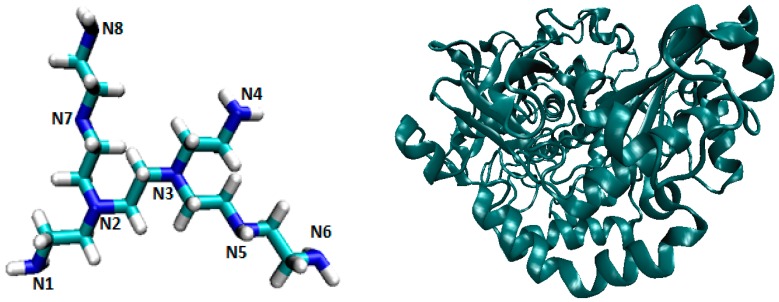
The ligand PEI_C14N8_07_B22 (**left**) and protein 3QVR (**right**, Glucose oxidase GOx).

Polyethylenimine (PEI) was intensely studied as a drug delivery system (DDS) in modern anti-cancer therapies [11,12]. PEI gained a large variety of uses, as published to date: in cosmetics as a thickener ingredient (viscosity adjuster), as a very effective polymer for neutralization of the excessive anionic charges (appearing in colloidal suspensions), as a metal chelating agent in corrosion inhibition, a copolymer in solid polymeric electrolytes for electrical energy storage devices, etc. [13,14]. However, there is a little information about the properties of GOx enzyme attached to PEI [15]. This is why, in this work, a molecular dynamics analysis was performed on a PEI structure (PEI_C14N8_07_B22, Figure 1, left) bound to the glucose oxidase enzyme 3QVR (Figure 1, right).

## 2. Results and Discussion

### 2.1. Docking Results

The values of binding free energy data were collected in the vicinity of the protein active site, where the ligand PEI_C14N8_07_B22 was docked; data are given in Table 1 and Table 2.

**Figure 2:**
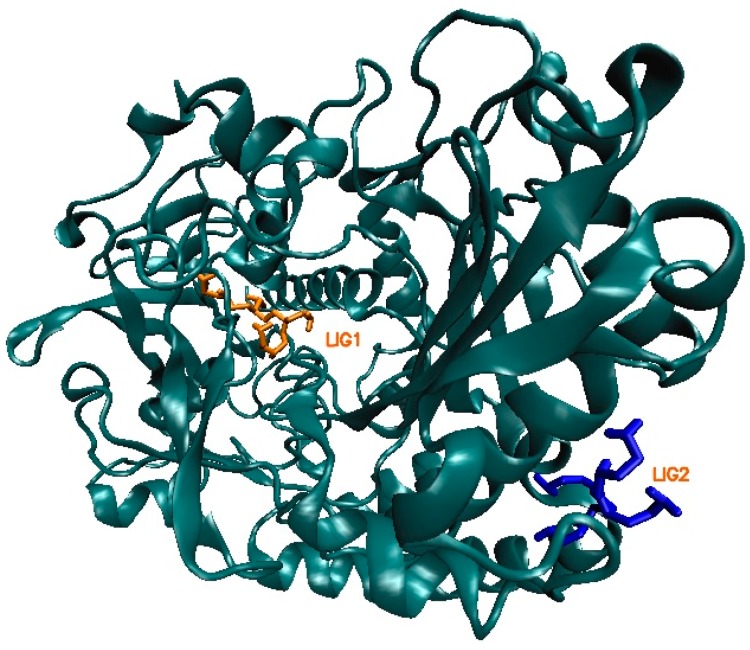
Two sites of the most important interactions ligand-enzyme (3QVR, Glucose oxidase): inside of the protein, named LIG1 and on its surface, named LIG2. As a ligand, the molecule PEI_C14N8_07_B22 was used (see Figure 1, **left**).

Based on the values of docked energy: −5.8 and −4.5 kcal/mol, two sites are differentiated on the protein body, which showed the best affinity to PEI (sites of type A, Table 1). In the first case, the ligand is bound to amino acids inside of the protein area (LIG1, Figure 2), while in the second case the binding takes place outside of the enzyme surface (LIG2, Figure 2). This is why the Molecular Dynamics will be performed for the first and second active places of this protein, separately. In addition, docking demonstrated that there are sites in the protein body that exhibit no interactions ligand–enzyme or even manifest repulsive characteristics (Table 2). The docking study gave information about the type and number of interactions between ligand and amino acid residuals of the enzyme. The results obtained during the docking procedure became the starting point in molecular dynamics study.

### 2.2. Molecular Dynamic Results

**Figure 3:**
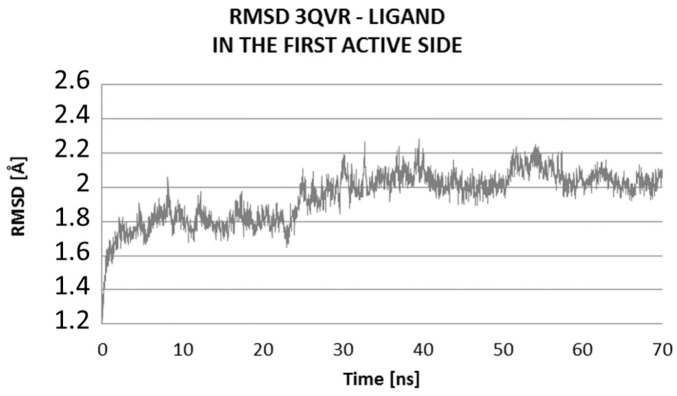
Distribution of RMSD (Root-Mean-Square Deviation) complex protein–ligand: Values characterizing the ligand interaction at the first active site (LIG1) of 3QVR (Glucose oxidase) enzyme, inside of the protein (Figure 2).

**Figure 4:**
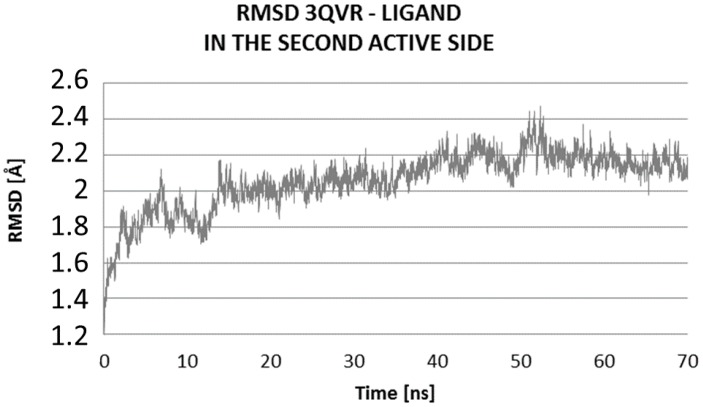
Distribution of RMSD complex protein–ligand: Values characterizing the ligand at the second active site (LIG2) of 3QVR (Glucose oxidase) enzyme (Figure 2).

The Molecular Dynamics (MD) simulations allow the study of enzyme-ligand interactions in the natural environment and in a large number of conformations of this complex, as well [16]. During MD, the time evolution of ligand PEI_C14N8_07_B22 lying in two active sites of the conformational space of 3QVR protein, inside (LIG1) and on its surface (LIG2) (Figure 2) was followed. The 60 ns of collected trajectories were used for structural analysis. In both cases, the first 10 ns of MD simulation show considerable fluctuations (as suggested by RMSD (Root-Mean-Square Deviation) values, Figure 3 and Figure 4), while in the remaining time of simulation, the trajectories seem stabilized, meaning the equilibrium stage was attained. In the case where the ligand interacts inside the protein body (LIG1, Figure 2), the system reached the equilibrium after 10 ns (Figure 3), while in the second case (ligand outside the enzyme surface (LIG2, Figure 2), after 15 ns (Figure 4).

**Figure 5:**
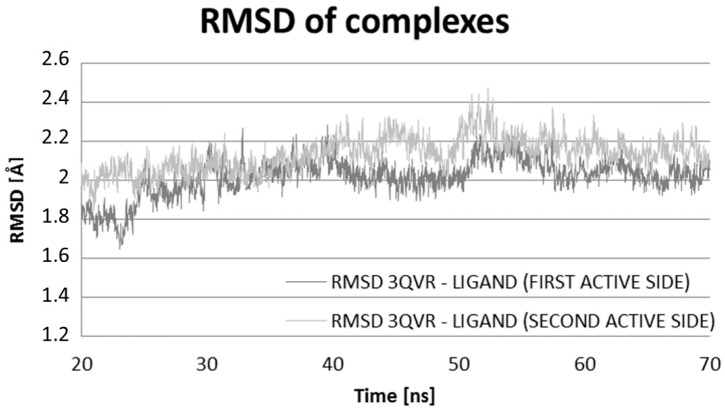
Distribution of RMSD complex protein–ligand: values characterizing the first active site (LIG1) of 3QVR enzyme and the second active site (LIG2) of 3QVR enzyme. The average values of RMSD (Å) of the two types of complexes for all trajectories (70 ns) with the standard deviations.

Comparing the RMSD values of the two systems, their average values and standard deviations, we can see that certainly they are similar, and the difference in average RMSD values is only 0.1 Å (Figure 5, Table 3).

**Figure 6:**
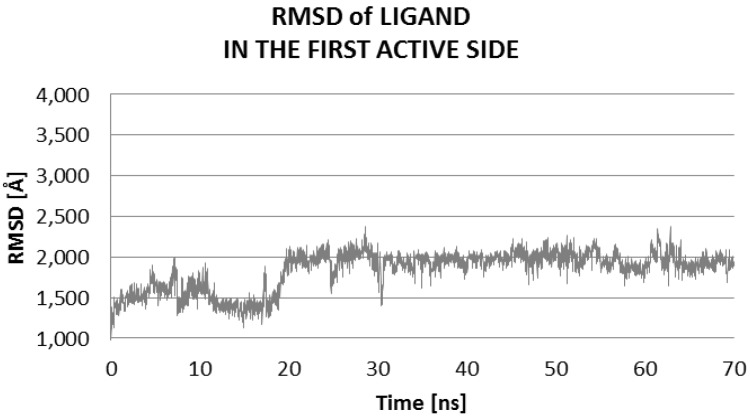
Distribution of RMSD of ligand: values characterizing the ligand interaction at the first active site (LIG1) of 3QVR enzyme, inside the protein (Figure 2).

At the first active side, inside of the protein, after 20 ns of MD (Molecular Dynamic), there is evidence of stabilization of RMSD (Root-Mean-Square Deviation) values of ligand, with poor fluctuations, from 1.6 to 2.0 Å (Figure 6). The average value of RMSD of ligand within the complex at LIG1 side is 1.84 ± 0.24, while for the complex with the ligand bound at LIG2 site, is higher, up to 2.78 ± 0.30 Å, suggesting a significantly higher mobility of ligand on the protein surface in comparison to the inside of enzyme (Table 3).

**Figure 7:**
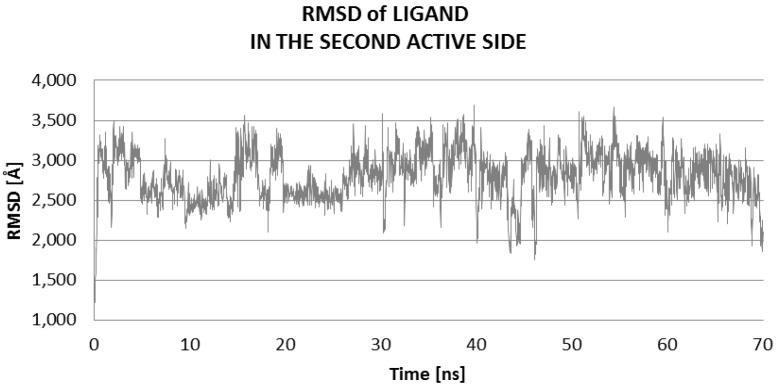
Distribution of RMSD of ligand: values characterizing the ligand interaction at the second active site (LIG2) of 3QVR enzyme, on the surface of protein (Figure 2).

Behavior of ligand on the surface of protein is similar during all trajectories. Here, the hydrogen bonds are weak, thus they are quickly broken and re-made, cyclically; such weak hydrogen bonds have a length of about 3 Å (Figure 7).

The high fluctuation of the values of RMSD of ligand on the surface of protein during MD was located in the domain of 2 Å to 3.5 Å (Figure 7).

**Figure 8:**
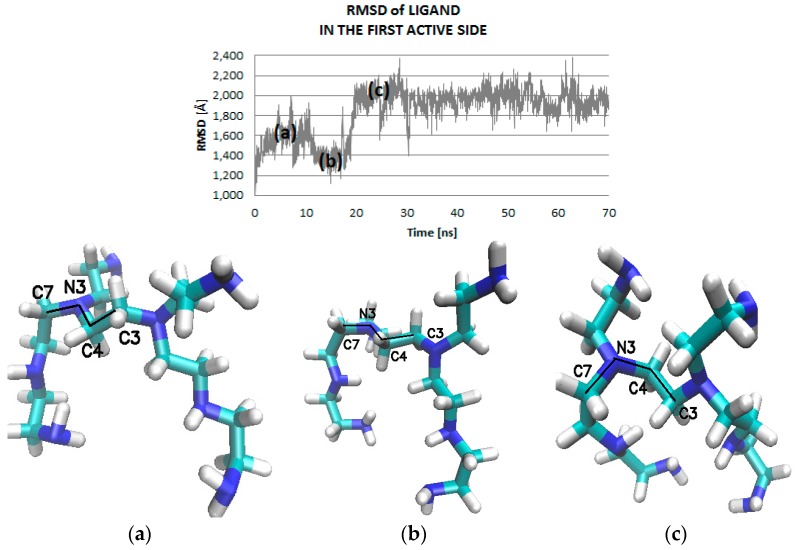
Distribution of values of the dihedral angle of ligand (C7-N3-C4-C3): values characterizing the ligand interaction at the first active site (LIG1) of 3QVR enzyme, inside the protein (Figure 2) during different periods of MD simulation: (**a**) first 10 ns; (**b**) 10–20 ns; (**c**) 20–70 ns.

**Figure 9:**
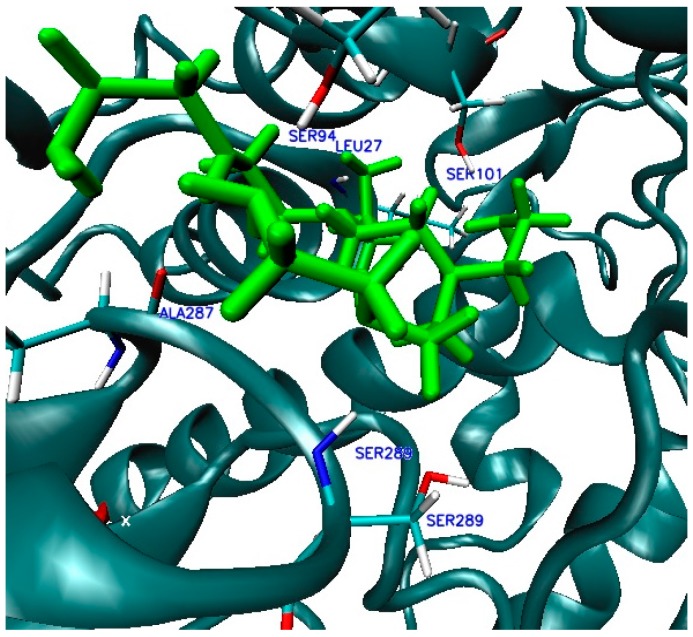
Interactions 3QVR enzyme-ligand PEI_C14N8_07_B22, at the first active site (LIG1).

**Figure 10:**
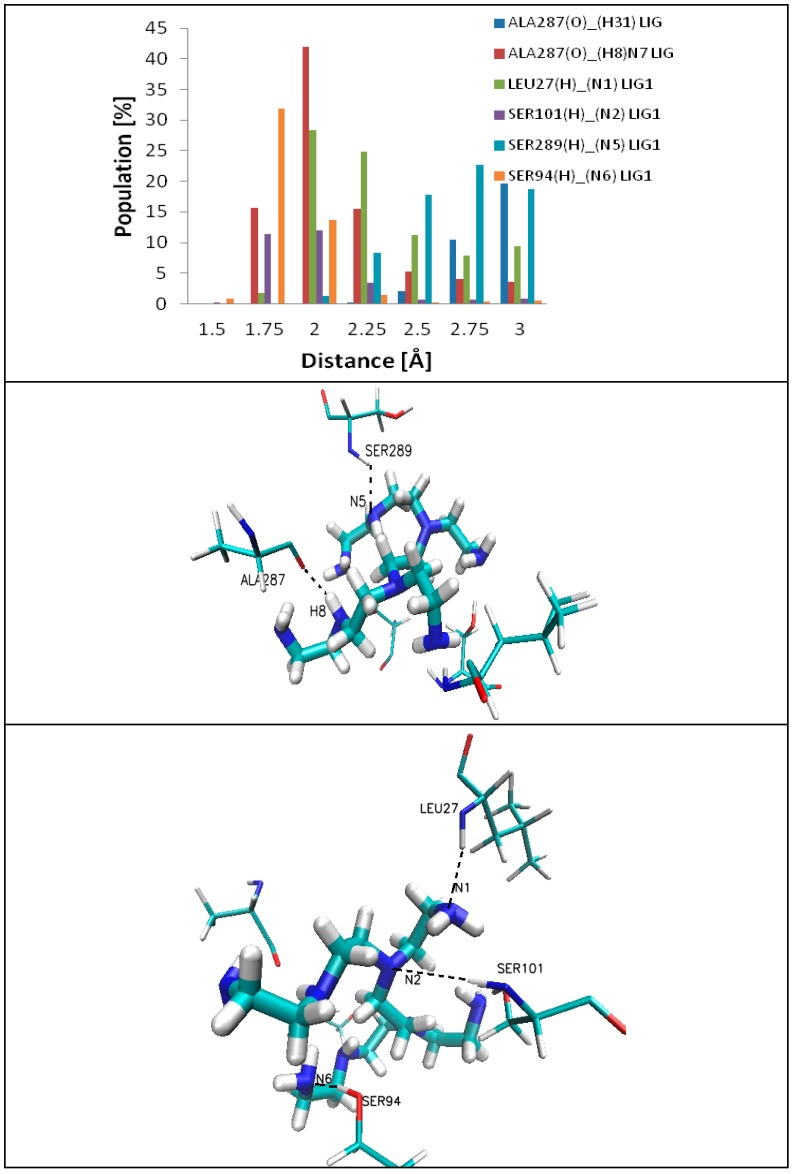
Distribution of the hydrogen bond lengths/interactions between PEI_C14N8_07_B22 and the amino acids at the LIG1 site of 3QVR (inside of protein), throughout the simulation time of MD.

**Figure 11:**
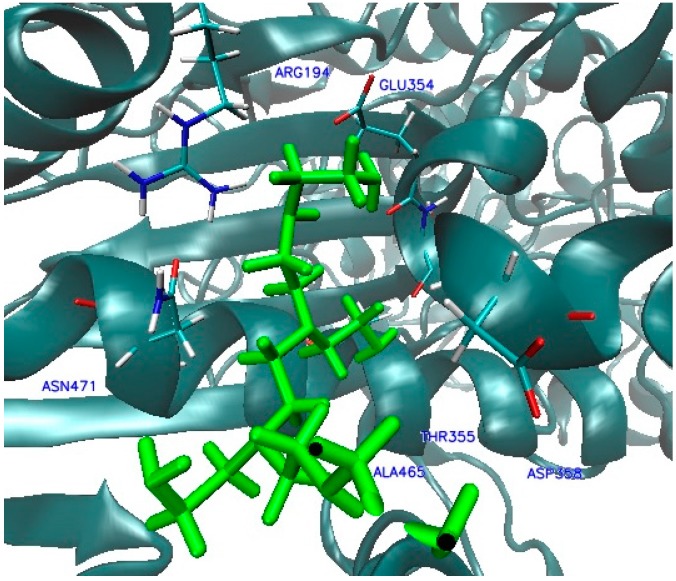
Interactions 3QVR enzyme-ligand PEI_C14N8_07_B22, at the second active site (LIG2).

**Figure 12:**
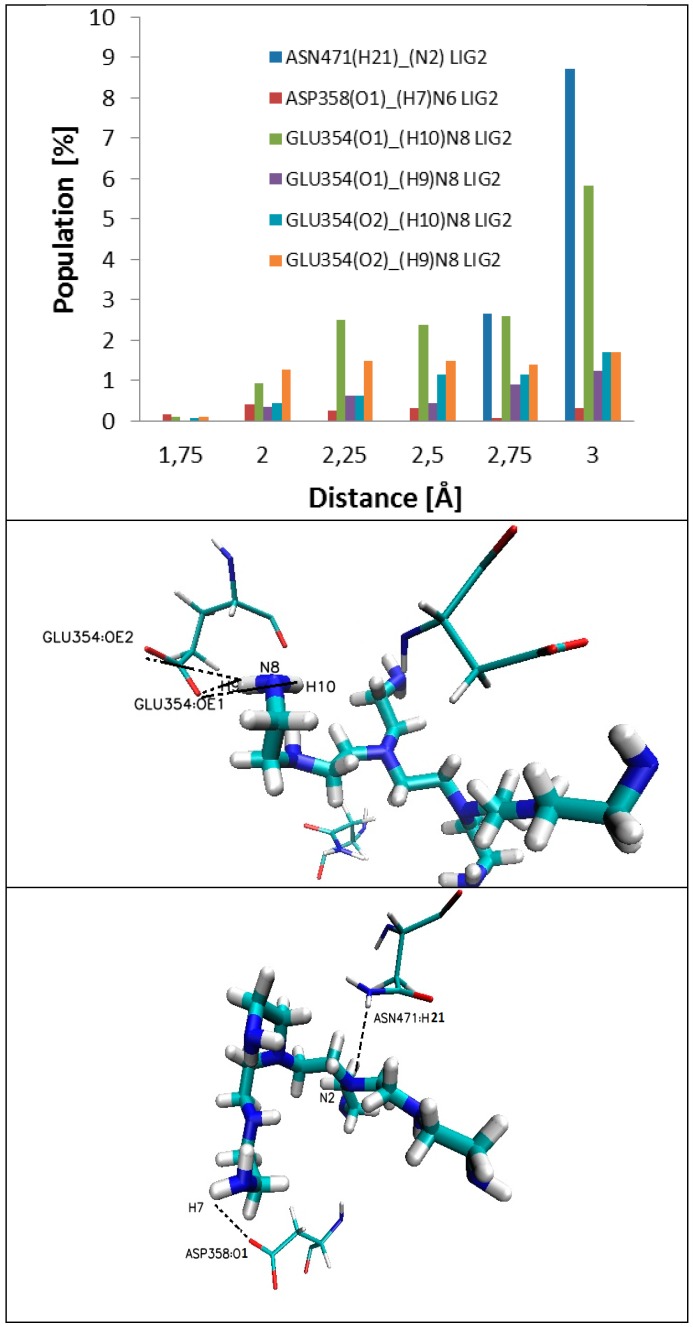
Distribution of the hydrogen bond lengths/interactions between PEI_C14N8_07_B22 and the amino acids at the LIG2 site of 3QVR (on the surface of protein), throughout the simulation time of MD.

The mobility of the ligand in the active site is correlated with changes in the dihedral angle values (C7-N3-C4-C3, Figure 8), with subsequent fluctuations in stabilization of hydrogen bonds and influence in the strength of hydrogen bonds (amino acid of enzyme (H)…..N(H_2_) ligand). After 20 ns of simulation, there appears a stabilization of the values of dihedral angle of ligand (C7–N3–C4–C3; Figure 6 and Figure 8, with an average value of −56 Å. The stabilization of RMSD values of ligand (Figure 8, Root-Mean-Square Deviation) is an indication of the creation of hydrogen bonds (HB) between the ligand and enzyme. Inside of the protein, six types of interactions (i.e., bonds) ligand–enzyme (Figure 9 and Figure 10) were found; they are of strong and medium strength (Figure 10). The complex formed on the protein surface also shows six types of interactions (Figure 11 and Figure 12), of a rather poor strength (Figure 12), favorable to hydrogen bond rupture.

In the case of complexes located inside the enzyme body (LIG1, Figure 2), the MD simulation (Molecular Dynamic) found five amino acids: ALA287, LEU27, SER101, SER94 and SER289, involved in the creation of six HB (Figure 9 and Figure 10) that stabilize the complex of enzyme 3QVR (Glucose oxidase) with PEI (polyethylenimine). In the case of complex located outside of the enzyme surface (LIG2, Figure 2), the first involved are ASN471, ASP358 and GLU354 (Figure 11) in the forming of the six HB (Figure 12).

**Figure 13:**
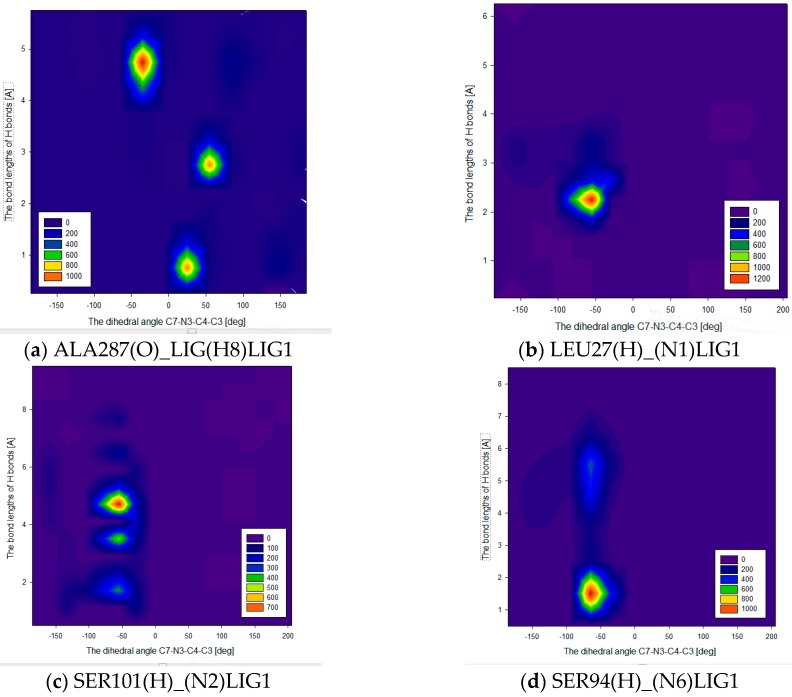
The length of hydrogen bonds at LIG1 (inside of protein): (**a**) ALA287(O)….(H8)LIG1; (**b**) LEU27(H)….(N1)LIG1; (**c**) SER101(H)….(N2)LIG1; (**d**) SER94(H)….(N6)LIG1 as a function of dihedral angle C7-N3-C4-C3 during all the time of MD simulation.

The hydrogen bonds Y ... H…X, established between a donor X and an acceptor Y, are classified function of the distance d(Y,H) between the acceptor Y and Hydrogen atom. There are “strong” HB, with d(Y,H) < 1.6 Å; “average/medium”, with 1.6 < d(Y,H) < 1.9 Å and “poor/low”, with d(Y,H) > 1.9 Å. With such a criterion, the protein 3QVR appeared to have a medium to low capacity of forming HB with the ligand PEI_C14N8_07_B22 [17] (Figure 9, Figure 10, Figure 11, Figure 12 and Figure 13).

In the first active site LIG1 (Figure 2) of the enzymes, there are five different amino acids: ALA287, LEU27, SER101, SER289 and SER94 (Figure 9 and Figure 10) that form HB with the ligand (PEI_C14N8_07_B22) as follows:

- ALA287(O)….(H31-PEI)LIG1, in 30% of all conformations, electrostatically interacts with the hydrogen atom H31 (of the ligand chain) to form a hydrogen bond of low strength;
- ALA287(O)….(H8-N7-PEI)LIG1, in 73% of all conformation, makes a strong hydrogen bond (Figure 11, Table 4).

For the interaction of PEI (polyethylenimine) with other aminoacids, located either at the first side (inside the enzyme) or at the second site (outside the enzyme surface), the reader can easily use Table 4 and Figure 10 and Figure 12.

In the case of the complex forming at the enzyme surface, the interactions ligand–enzyme appear relatively late, during MD simulations, (after about 30 and 40 ns) and all of these impacts have medium and low strength. In addition, the formed bonds tend to disappear and re-appear during MD simulation (Figure 11 and Figure 12, Table 4).

Note that the appearance/disappearance of hydrogen bonds is closely related to the values of dihedral angle C7-N3-C4-C3 of the ligand PEI_C14N8_07_B22 (Figure 8 and Figure 13) in the complex at LIG1. In the case of ALA287(O)….LIG(H8)LIG1, while the angle C7-N3-C4-C4 reaches the value +50 degree [deg], the strongest hydrogen bonds (with lengths around 1.65 Å—Figure 13a) are formed. However, the increase of the dihedral angle values from 50 up to 100 degree [deg] is associated with an increased length of the hydrogen bonds, from 2.5 Å to 3 Å, meaning a decrease of HB strength with the appearance of hydrogen bonds of medium and low strength. For the interaction LEU27(H)….(N1)LIG1, the value of the dihedral angle C7-N3-C4-C4 is about −50 degree [deg] and the hydrogen bond reaches a length of about 2.2 Å (Figure 13b). Here, corresponding to the interval −25 to −100 degree [deg] of dihedral angle, there appear hydrogen bonds of length ranging from 1.8 Å to 2.5 Å. The bond SER101(H)……(N2-PEI)LIG1 periodically reaches a length of 1.8 Å (Figure 13c). In the case of SER94(H)….(N6)LIG1, such interactions are characterized as strong and medium while the values of dihedral angle ranged from −50 to −90 degree [deg] (Figure 13d).

As one can see, the stabilization of ligand–protein complexes is related to the strength and number of hydrogen bonds created between them. This interaction can be characterized by the values of calculated enthalpy contribution of Gibbs Free Energy; in the two cases of complex PEI-enzymes, located inside of the protein (LIG1) and on its surface (LIG2), the values of enthalpy are −12.486 ± 8.7321 and −13.7496 ± 6.7161, respectively.

## 3. Materials and Methods

During the docking simulation, the whole area of the enzyme was mapped, by changing the parameters of grid box step by step; the crystal structures of 3QVR were used, as downloaded from the Brookhaven Protein Database (PDB) [18,19]. A Molecular Dynamics (MD) procedure was applied to the two best-docked enzyme-ligand (3QVR-PEI_C14N8_07_B22) complexes. The ligand structure was characterized by using the Amber force field parameters; the atomic charges were calculated according to the Merz–Kollmann scheme, via the RESP procedure [20] at HF/6-31G* level of theory. Each system was neutralized and immersed in a periodic TIP3P (Transferable Intermolecular Potential 3P) water box (Jorgensen et al. 1983). The considered systems were heated up to 300 K by 100 ps of initial MD simulation, while the temperature was controlled by the Langevin thermostat (IN&NY, USA) [21]. The periodic boundary conditions and SHAKE algorithm [22] were applied to 70 ns of MD simulation, the first 10 ns of the simulation time being used for the equilibration of the system; the next 60 ns of trajectory were used in the effective analysis of interaction between the considered subunits. Structural analysis was performed by the VMD (Visual Molecular Dynamic) package [23]. The energetic characterization of the interaction ligand–enzyme active site was obtained by the Molecular Mechanic/Poisson-Boltzmann Surface Area (MMPBSA) method [24]. In all MD simulations, the AMBER 11 package (San Francisco, CA, USA) [25] was used.

## 4. Conclusions

Glucose oxidase (GOx) is an enzyme that has found several commercial applications in chemical and pharmaceutical industries including biosensors that use this enzyme immobilized onto different nanomaterials and polymers such as polyethylenimine (PEI). The present data for the first time describe a more specific and oriented interaction of glucose oxidase and PEIs which can serve in different bioconjugation processes, and, at the same time, this part of the work is a proposition to continue the incipient study with other shapes/sizes of PEI. The problem of GOx immobilization on PEI is to retain the enzyme native activity despite its immobilization onto the polymer surface. Therefore, an MD study of the PEI ligand (C14N8_07_B22) and GOx enzyme (3QVR) was performed to investigate the stability of the complex C14N8_07_B22-3QVR and the affinity of the PEI ligand to the docking sites of GOx. Analysis of the dynamic behavior of complexes formed by the ligand PEI_C14N8_07_B22 with 3QVR inside of the enzyme body and on its surface, respectively, revealed important differences in their structural and energetic characteristics. For the both complexes, conformations stabilized by hydrogen bonds were observed during the docking stage. The docking procedure showed two sites of major interaction of the protein with the polymer PEI: (LIG1), of −5.8 kcal/mol and (LIG2), of −4.5 kcal/mol, located inside the enzyme and on its surface, respectively. Docking also found domains of protein that exhibit no interactions with the ligand or have even repulsive characteristics. The results obtained during docking became the starting point in molecular dynamics simulation. The RMSD (Root-Mean-Square Deviation) values of systems, their average values and standard deviations show that these two systems (LIG1 and LIG2) are quite similar. In the first active side, inside of the protein, after 20 ns of MD (Molecular Dynamic), stabilization values of RMSD of ligand appears, with poor fluctuations from 1.6 to 2.0 Å. The average values of RMSD of ligand show significantly higher mobility of PEI (polyethylenimine) on the surface of protein (at LIG2) compared to that inside of enzyme (at LIG1). The mobility of the ligand inside of the protein is correlated with changes in the values of dihedral angle (C7-N3-C4-C3). The hydrogen bonds (i.e., interactions) formed between enzyme and ligand, are shorter (i.e., stronger) inside of the protein compared to HB on the protein surface. The lengths of the hydrogen bonds ligand–enzyme created on the surface of protein are long and hence HB and the corresponding interactions are weak.

## Figures and Tables

**Table 1:** Lamarckian genetic algorithm docked state—Binding energy of ligand PEI_C14N8_07_B22 to the active sites of type A of 3QVR (Glucose oxidase) during the nine explored conformations.

Ligand Binding Site	1	2	3	4	5	6	7	8	9	Best Docking Energy (kcal/mol)
LIG1	−5.8	−5.8	−5.6	−5.6	−5.6	−5.5	−5.5	−5.5	−5.5	−5.8
LIG2	−4.5	−4.3	−4.2	−4.2	−4.2	−4.2	−4.2	−4.1	−4.1	−4.5
LIG3	−3.8	−3.8	−3.7	−3.6	−3.6	−3.6	−3.6	−3.6	−3.5	−3.8
LIG4	−3.9	−3.4	−2.7	−1.9	−1.5	−1.2	×	×	×	−3.9

**Table 2:** Lamarckian genetic algorithm docked state—Binding energy of ligand PEI_C14N8_07_B22 to the active sites of type B of 3QVR during the nine explored conformations.

Ligand Binding Site	1	2	3	4	5	6	7	8	9	Docked Energy (kcal/mol)
1	0.0	0.0	0.0	0.0	0.0	0.0	0.0	0.0	0.0	0.0
2	−0.6	−0.4	−0.2	0.0	0.0	0.1	0.2	0.3	0.4	−0.6
3	0.8	1.6	2.4	2.7	3.0	×	×	×	×	0.8

**Table 3:** Average RMSDs for the ligand and for the amino acids comprising the active site, across the full MD simulation.

	Ligand Close to LIG1 of 3QVR	Ligand Close to LIG2 of 3QVR	Complex Ligand–Enzyme at LIG1 Site	Complex Ligand–Enzyme at LIG2 Site
RMSD (Å)	1.84	2.78	1.95	2.05
SD	0.24	0.30	0.14	0.16

SD = standard deviation.

**Table 4:** The length distributions of the most common hydrogen bonds (electrostatic interactions) occurring between PEI_C14N8_07_B22 and selected amino acids from the LIG1 site (inside of the enzyme; left side of the table) and from the LIG2 site (on the surface of enzyme; right side of the table) of 3QVR during MD simulations.

Hydrogen Bonds at LIG1	Hydrogen Bond Length (Å)	Population (Among All Conformations) (%)	Hydrogen Bonds at LIG2	Hydrogen Bond Length (Å)	Population (Among All Conformations) (%)
ALA287(O)_(H31)LIG1	2.25	0.03	ASN471(H21)_(N2)LIG2	2.75	2.7
	2.5	2.11	ASP358(O1)_(H7)N6LIG2	1.75	0.17
	2.75	10.48		2	0.42
	3	19.62	GLU354(O1)_(H9)N8LIG2	2	0.4
ALA287(O)_(H8)N7LIG1	1.75	15.7		2.25	0.6
	2	41.9		2.5	0.4
	2.25	15.5		2.75	0.9
	2.5	5.2		3	1.3
	2.75	4.0	GLU354(O1)_(H10)N8LIG2	1.75	0.1
	3	3.6		2	0.9
LEU27(H)_(N1)LIG1	1.7	1.8		2.25	2.5
	2	28.3		2.5	2.4
	2.25	24.8		2.75	2.6
	2.5	11.2		3	5.8
	2.75	7.8	GLU354(O2)_(H9)N8LIG2	1.75	0.1
	3	9.5		2	1.3
SER101(H)_(N4)LIG1	1.5	0.14		2.25	1.5
	1.7	11.31		2.5	1.5
	2	11.94		2.75	1.4
	2.25	3.48		3	1.7
	2.5	0.63	GLU354(O2)_(H10)N8LIG2	1.75	0.09
	2.75	0.60		2	0.45
	3	0.86		2.25	0.63
SER94(H)_(N6)LIG1	1.5	0.9		2.5	1.16
	1.7	31.9		2.75	1.16
	2	13.7		3	1.7
	2.25	1.4			
	2.5	0.3			
	2.75	0.4			
	3	0.5			
SER289(H)_(N5)LIG1	2	1.2			
	2.25	8.3			
	2.5	17.7			
	2.75	22.7			
	3	18.8			

## Supplementary Materials

Supplementary File 1
