# Supplementary figures and images for: Molecular Dynamic Studies of the Complex Polyethylenimine and Glucose Oxidase
Source: Int J Mol Sci. 2016 Oct 27;17(11):1796. doi: 10.3390/ijms17111796 (PMC5133797; doi:10.3390/ijms17111796)

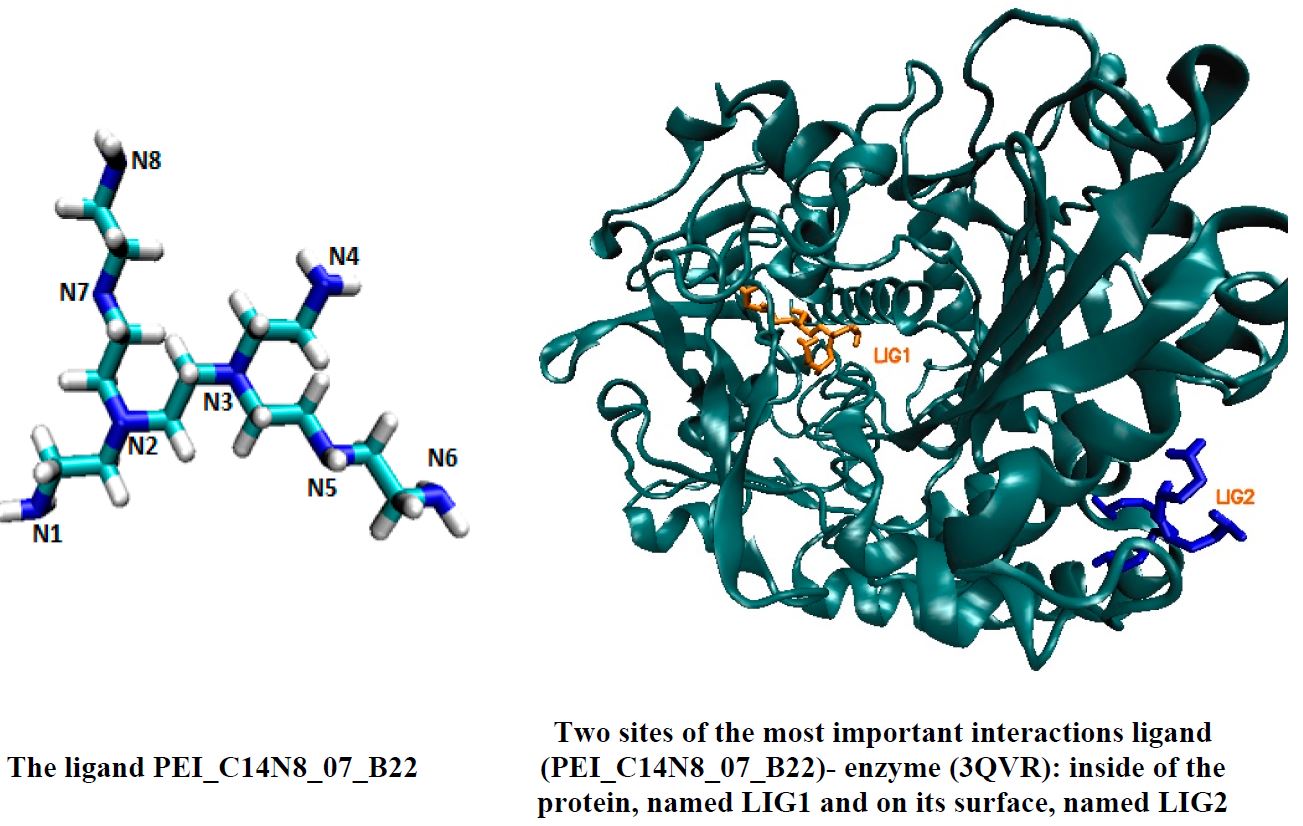

Supplement: Supplementary File 1 [file ijms-17-01796-s001.jpg]
